# Immune complexes in chronic Chagas disease patients are formed by exovesicles from *Trypanosoma cruzi* carrying the conserved MASP N-terminal region

**DOI:** 10.1038/srep44451

**Published:** 2017-03-15

**Authors:** Isabel María Díaz Lozano, Luis Miguel De Pablos, Silvia Andrea Longhi, María Paola Zago, Alejandro Gabriel Schijman, Antonio Osuna

**Affiliations:** 1 Departamento de Parasitología, Grupo de Bioquímica y Parasitología Molecular, Campus de Fuentenueva, Universidad de Granada, 18071 Granada, Spain; 2 Centre for Immunology and Infection (CII), Biology Department, University of York, York, UK; 3 LabMeCh, INGEBI-CONICET, Buenos Aires, Argentina; 4 Instituto de Patología Experimental (IPE) CONICET-UNSa, Argentina

## Abstract

The exovesicles (EVs) are involved in pathologic host-parasite immune associations and have been recently used as biomarkers for diagnosis of infectious diseases. The release of EVs by *Trypanosoma cruzi*, the causative agent of Chagas disease, has recently been described, with different protein cargoes including the MASP multigene family of proteins MASPs are specific to this parasite and characterized by a conserved C-terminal (C-term) region and an N-terminal codifying for a signal peptide (SP). In this investigation, we identified immature MASP proteins containing the MASP SP in EVs secreted by the infective forms of the parasite. Those EVs are responsible for the formation of immune complexes (ICs) containing anti-MASP SP IgGs in patients with different (cardiac, digestive and asymptomatic) chronic Chagas disease manifestations. Moreover, purified EVs as well as the MASP SP inhibit the action of the complement system and also show a significant association with the humoral response in patients with digestive pathologies. These findings reveal a new route for the secretion of MASP proteins in *T. cruzi*, which uses EVs as vehicles for immature and misfolded proteins, forming circulating immune complexes. Such complexes could be used in the prognosis of digestive pathologies of clinical forms of Chagas disease.

Chagas disease is an endemic disease in 21 South and Central American countries, affecting around 6–7 million people worldwide^1^, with 50.000 to 200.000 news cases each year^2^. This infection is caused by *Trypanosoma cruzi*, an intracellular flagellated protozoan parasite belonging to the group Kinetoplastida, which is transmitted in nature by bloodsucking triatomine vectors^1^. Chagas disease was considered as exclusively endemic to Central and South America until the two decades of the last century, however, the migration flows of infected individuals has extended the disease to non-endemic countries such as USA, Canada, Spain, Italy, Switzerland, Sweden, Japan or Australia^3^,^4^. The life cycle of *T. cruzi* begins when the infective metacyclic trypomastigote forms are released in the faeces of the insect vector. These forms will infect surrounding epithelial cells, where they differentiate into intracellular amastigotes which after several rounds of replication will differentiate into bloodstream forms which will be dispersed through the bloodstream, infecting different organs and tissues of the host cell body. In the majority of cases, the acute phase of the disease produces similar symptoms to flu (fever, headache or muscle pain) with high numbers of circulating bloodstream trypomastigotes^5^. The chronic phase is characterized by the presence of few or barely detectable bloodstream forms and the presence of nests formed by intracellular forms mainly inside cardiac and/or gastrointestinal tissues^6^. According to WHO, during the chronic phase 20 to 30% of infected patients will suffer irreversible cardiovascular, gastrointestinal, and/or neurological problems^7^.

*T. cruzi* produces a strong polyclonal humoral immune response in the acute phase, partially due to the recognition of parasite surface proteins by B cell receptors and macrophage proteins along with the production of proinflammatory cytokines leading to the persistence of the parasite inside the host^8^,^9^. Among the most antigenic proteins in *T. cruzi,* three multigene protein families are present on the parasite surface, namely: *trans*-sialidases (TSs), mucins and mucin-associated surface proteins (MASPs)^10^,^11^, that are encoded by a large number of genes bearing repeated sequences (RSs) or sequences repeated in tandem (TRSs)^12^.

The MASP family is composed of 5′ and 3′ conserved regions in all *T. cruzi* strains sequenced to date, codifying for a signal peptide (SP) and a C-terminal region, which serve as potential signals for their location at the surface of the parasite^13^,^14^,^15^,^16^. The highest RNA and protein expression of *masp* genes occurs in the infective forms being also secreted to the extracellular medium by metacyclic and bloodstream trypomastigote forms during the infection process^16^,^17^,^18^. The humoral response to the MASP C-term region in a pool of sera from patients with different chronic Chagas disease pathologies tested positive for all of them, being also able to block the action of lytic antibodies in a pool of sera from patients with digestive pathologies^19^.

During the initial phase of infection, *T. cruzi* is capable of evading the lysis mediated by the complement system due to the inactivation of the C3b/C4b component of alternative and classical complement pathway by direct binding to glycoproteins such as gp160, gp58/68 and calreticulin (TcCRT) on the surface of parasite^20^,^21^,^22^. All these mechanisms conclude with the progression to the chronic phase of the disease (~60 days post-infection) with an hypergammaglobulinemia increase in murine models characterized by a rise of the IgM, IgG1, IgG3 and IgG2b and IgG2a isotypes, with a predominance of the last which increases up to ten times its normal value^23^,^24^, the presence of immune complexes (ICs) in patients with Chagas disease has been reported since the 1980s^25^,^26^,^27^,^28^. These immune complexes are formed in the chronic phase of the illness by immunoglobulins and secreted antigens of the parasite. Despite of the potential role of ICs in the pathogenesis of Chagas disease, there is a scarcity of studies directly addressing this issue in comparison to studies of other parasites such as *Leishmania spp.*^29^. One recently published proteome of circulating ICs in chronic Chagas disease patients has shed light onto the composition of these ICs which appear to be formed by proteins belonging to *T. cruzi* surface families involved in processes of adhesion or invasion of host cells among others^30^.

An important part of this antigenic material released by the parasite is packaged into “Exovesicles” (EVs). EVs are defined as membrane-bound particles enclosed by a lipid bilayer and released by cells into the extracellular environment^31^. There are two different EV populations in biological fluids (blood, urine, saliva, etc.), one described as exosome-like EVs, with a size of 70–90 nm and originating from multivesicular bodies (MVBs)^32^, and the second described as ectosome-like EVs, with a size varying between 130–140 nm and released by budding membranes originated from vesicular transport^33^. EVs could have biomedical applications due to their size and composition as biomarkers of disease, such as has been reported in cancer, renal disorders and pathogenic infections where they have a central role in cell to cell communication^34^,^35^,^36^,^37^,^38^,^39^. The release of EVs by pathogens and their role in disease progression by modulating the host immune response has been demonstrated in several studies^40^,^41^,^42^,^43^,^44^. In *T. cruzi*, the existence of proteins belonging to surface proteins within these EVs, including the highly conserved C-term region of the MASP family^19^ has been previously reported^45^,^46^,^47^,^48^. Additionally, EVs could be also produced by the host cell in response to the presence of the parasite. For instance, metacyclic trypomastigotes induce a 3 fold increase in the number of plasmatic membrane vesicles (PMVs) from infected host cells (blood and lymphoid cells) as it has been demonstrated *in vitro* and *in vivo* assays^49^. These released PMVs are capable of inactivating the classical and lectin-complement pathway avoiding the death of the parasite.

In this study, the presence of MASP SP in EVs as well as its humoral response is investigated. Additionally, the presence of this non-canonical secreted peptide is also described as a part of the circulating ICs in Chagas disease patients.

## Materials and Methods

### Human serum samples

All protocols involving human subjects were approved by the Medical Ethics Committee of Ramos Mejía Hospital from Buenos Aires (www.bioeticarm.org 19/2/14) and Facultad de Ciencias de la Salud, Universidad Nacional de Salta and San Bernardo Hospital from Salta, Argentina (N°416–15). A total of 123 enrolled patients provided written informed consent prior to initiation of study activities and the experiments were performed in accordance with the approved guidelines, who had at least two immunological techniques were considered positive for Chagas disease. All study participants provided written informed consent prior to initiation of study activities and the experiments were performed in accordance with the approved guidelines. The sera were classified according to the symptomatology of the patients: ASYMP: chronic Chagas disease patients who have normal complementary studies (n = 31); CARD: chronic Chagas heart disease patients who have abnormal complementary studies such as ECG and/or echocardiography, who are subclassified according to the clinical form in CARD-Arr, patients with arrhythmias and/or conduction disorders without heart failure (n = 36) and CARD-ICC patients with heart failure cardiomegaly and/or ventricular dysfunction with or without arrhythmias (n = 28) (see Supplementary Table S1); DIG: chronic Chagas disease patients with gastrointestinal symptoms and evidence of megacolon (n = 28).

### Parasites and cell cultures

The parasite strain used was PAN4 (DTU TcI), isolated from a patient in Arraijan, Panama in 2004. The flagellate epimastigote forms (E) were cultured at 28 °C in MTL medium supplemented with 10% heat-inactivated foetal calf serum (IFCS) as described Ruiz-Perez *et al*.^50^. The infective trypomastigote (T) and amastigote (A) forms were obtained from infected culture Vero cells as previously described^51^,^52^.

Only T and A form samples with at least 95% purity by Giemsa staining were used in further experiments.

### Bioinformatics analysis and peptide synthesis

To design a synthetic peptide representative of the consensus sequence of MASP SPs, we used as a query for BLAST searches the conserved SP sequence of the MASP52 protein^17^ annotated in Tritrypdb database (http://tritrypdb.org/tritrypdb/). Then, the SP sequences of 250 homologous MASP proteins were analysed by Multiple Em for Motif Elicitation 4.3.0. (MEME) program (http://meme-suite.org/tools/meme) to obtain the final MASP SP consensus sequence (MAMMMTGRVLLVCALCVLWSVAADG). Finally, the peptide was synthetized by LifeTein (USA, LLC) with four branches bounds by its Lysine residues (see Supplementary Fig. S1).

### EVs purification

After the purification of the different *T. cruzi* life cycle stages described above, the A and T forms were incubated for 12 h at 37 °C whilst E forms were incubated at 28 °C in the respective culture media (RPMI 1640 supplemented with 25 mM HEPES pH 7.4 and 10% free-EVs IFCS for T and E forms, and the same medium at pH 6.4 for A forms). The infections of Vero cells with infective forms were performed as described by De Pablos *et al*.^17^.

After 12 h post-infection, the parasite-host cell interaction medium (IM) was removed and the EVs were purified as previously described^19^. For the interaction medium the first pellet obtained after ultracentrifugation at 100000 × g 1 h was conserved and called ectosomes, and the final pellets obtained corresponded with exosomes.

### Polyclonal antibodies production

Animal experiments were performed in accordance with institutional guidelines (Spanish government regulations (Real Decreto RD1201/05)) and European Union guidelines (European Directive 2010/63/EU) and were approved by the local authorities (Resolution n° 522 of the Ethics Committee of the University of Granada). The production of polyclonal antibodies was done by intraperitoneal injection of Wistar rats or Balb/c mice with 100 μg and 20 μg of peptide MASP SP per dose, respectively. Before the first step of immunization, a bleed for pre-immune control serum was done. The next boosts were performed 2 and 4 weeks later with the same amount of peptide and the animals were finally bled 2 weeks after the last boost. The antibody titters of the anti-MASP SP sera were checked by indirect enzyme-linked immunosorbent assay (ELISA) in Amino immobilizer multiwells plates (Nunc, Thermo fisher) coated with 5 μg of the synthetic peptide/well in 0.1 M bicarbonate coating buffer (pH 9.6). Sera with titters higher than 1:6400 were pooled and stored at −80 °C diluted 1:1 with glycerol (grade for Molecular Biology, Sigma) until use and named as anti-MASP SP immune sera.

To obtain specific polyclonal antibodies against *T. cruzi,* soluble extract proteins from epimastigotes forms were immunized in rabbits (320 μg/doses) following the protocol described above. Proteins samples were purified from T forms EVs were fixed by 2.5% glutaraldehyde solution in cacodylate buffer at pH 7.2 for 24 h at 4 °C, with continuous agitation. The free reactive aldehyde groups were blocked in a molar excess of glycine solution in carbonate buffer at pH 9.4. Finally, the proteins were dialyzed in Slide-A-Lyzer™ G2 Dialysis Cassettes (Thermo Scientific) with 2.000 MWCO pore size and 0.5 ml capacity. The dialysis was performed following manufacturer’s instructions for 24 h at 4 °C in 0.1 M acetate buffers at pH 7. A total of four dialysis changes were done. Next, the proteins were lyophilized and resuspended in PBS for immunization following the protocols described above in mice Balb/c strain. Sera with titters higher than 1:1600 were pooled and stored at −80 °C diluted 1:1 with glycerol until use and labelled as anti-EVS immune sera.

### SDS-PAGE and Western blotting

The EVs pellet from T forms together with ectosomas and exosomes from T forms and Vero cells interaction medium, were treated with RIPA buffer (50 mM Tris-HCl pH 7.4, 1% NP-40, 0.5% Na-deoxycholate, 0.1% SDS, 150 Mm NaCl, 2 Mm EDTA, 50 Mm NaF) and disrupted by sonicating the samples three times on ice (at intervals of 15 s for a total of 2 min, with a 15 s pause) in a Branson SLP Sonicator. The EVs protein concentration was determined using Micro BCA^TM^ Protein Assay Kit (Thermo Scientific ^TM^). The samples were mixed in 1:1 volume with 2x Laemmli sample buffer^53^ with 10% (v/v) β-mercaptoethanol (Sigma) and heated at 95 °C for 10 min. Next, the samples were separated by SDS-PAGE in Mini-Protean TGX gel 4–20% (Bio Rad) 30 min 200 V and transferred to polyvinylidene difluoride (PVDF) 0.2 μm size of pore (Bio-Rad) in a Transblot turbo System (Bio Rad) according the method described by Towbin et al (1979)^54^. Next to the transference, the membranes were incubated in blocking buffer (PBS, 0.5% (v/v) Tween-20 (PBS-T) and 2% (w/v) milk powder) overnight at 4 °C. The membranes were then incubated with anti-MASP SP at dilution 1:500 in blocking buffer for 2 h RT. After three washed steps with PBS-T, the membranes were incubated with secondary antibody conjugated with horseradish peroxidase (HRP), polyclonal goat anti-rat immunoglobulin (SIGMA) at dilution 1:5000 in PBS-T. The reaction was developed using Western Clarity™ Western ECL Substrate (Bio-Rad).

### Scanning Electron Microscopy (SEM)

For scanning electron microscopy the cells were cultured over glass coverslips and infected with the T forms as described above. After two hour of interaction with the parasite, the infected cells were fixed with 2.5% glutaraldehyde in cacodylate buffer with 0.1 M saccharose and maintained in the fixative solution for 24 h at 4 °C. Next, the samples were dehydrated in a graded series of ethanol, desiccated using a critical point dryer (LEICA EM CPD 300) and then evaporated with high vacuum carbon coater (EMITECH K975X). The cells were finally coated with Carbone for 3 min and observed microscope in a ZEISS Supra 40VP high-resolution SEM.

### Transmission Electron Microscopy (TEM)

For Transmission Electron Microscopy T and E forms obtained from the culture were washed by centrifugation in PBS and fixed for 2 h at RT with 2.5% glutaraldehyde in cacodylate buffer with saccharose (0.1 M) pH 7.4 and then incubated at 4 °C for 24 h. After one step of centrifugation (300 × g, 10 min) to remove the fixative solution, the parasites were post-fixed for 2 h in 2% osmium tetroxide plus 1% potassium ferrocyanide in 0.1 M cacodylate buffer at RT. The fixed forms of *T. cruzi* were embedded in epoxy resin after having been dehydrated. Ultrathin 50–70-nm sections were cut and collected onto copper grids (300 mesh), stained with uranyl acetate and lead citrate and then examined under a Carl Zeiss SMT LIBRA 120 PLUS TEM.

### Confocal Microscopy

The immunofluorescence assays for the purified E, T and A forms of the parasites were performed in 10 well- “PTFE” Printed Slides (Electron Microscopy Sciences). After washing the parasites in PBS, these were fixed in 4% PFA (4% paraformaldehyde in PBS 0.1 M) solution for 30 min. Next, the parasites were washed three times in PBS and permeabilized with citric acid (10 mM), 1% NP-40 solution for 5 min at 95 °C. Then, the slides were washed with PBS and blocked for 1 h in PBS, 1% BSA at pH 7.2. The different samples were incubated with specific immune sera (anti-MASP SP developed in rat or anti-EVs Trypo developed in mouse) for 1 h at RT at dilution 1:50 in PBS. The samples were then incubated for 1 h, with secondary antibody anti-rat Alexa-fluor 633 dye (4 μg/ml) for the detection of anti-MASP SP and anti-mouse FITC (SIGMA) for anti-EVs Tripo (4 μg/ml). For DNA staining, the samples were treated for 10 min in DAPI solution (10 μg/ml) (4′,6-Diamidine-2′-phenylindole dihydrochloride, SIGMA). In order to preserve the fluorescence, the samples were immersed in mounting medium (Ibidi Mounting Medium, Ibidi).

As a control for all experiments, pre-immune sera from rats and mice were used. The fluorescence image was observed in Leica DM5500B confocal laser microscope.

### Immunogold labelling studies

The pellets with the purified EVs were fixed and treated as previously described^19^. The grids, containing the ultrathin section of the EVs were blocked in a blocking solution containing 1% (w/v) gelatine from cold-water fish skin (SIGMA) prepared in PBS and incubated for 10 min at RT, then the grids were placed upon of 0.02 M glycine in PBS and finally the blocking procedure concluded with incubation in a solution of 1% albumin from chicken egg white (Sigma) for a total of 15 min at RT.

The grids were placed upon 20 μl for 90 min at RT 1:20 dilution with anti-Clathrin antibodies (SCBT) as EVs putative marker control or anti-MASP SP. The grids were then washed five times in PBS for 25 min. Next, Gold-labelled (10 nm) secondary antibodies were used at 1:20 dilution under the same conditions. Finally, the grids were washed in PBS for 25 min in distilled water and contrasted with 2% (v/v) uranyl acetate solution. EM observations were done in Carl Zeiss SMT LIBRA 120 PLUS TEM.

### Depletion of immune complexes (ICs)

One aliquot of each serum sample was pre-treated for the disassembly of the immune-complexes (ICs). To do so, 20 μl of each serum were diluted in Glycine buffer (0.1 M) pH 4.5 at a dilution of 1:100 and stirred in an orbicular shaker for 15 min at RT. After the treatment, all aliquots of diluted sera were centrifuged at 2000 × g 30 min to remove cell debris. The supernatant was transferred to a new tube and centrifuged at 17000 × g for 30 min. Finally, the supernatant was ultra-centrifuged in microtubes (Hitachi No 1508) at 100000 × g 2 h at 4 °C in an ultracentrifuge (Sorwal WX80) with fixed angle rotor (Fiberlite™ F50L-24 × 1.5). The pellet was washed three times with PBS by ultracentrifugation under the same conditions as described above. To check the correct depletion of ICs from sera, the protein concentration was determined using the Micro BCA^TM^ Protein Assay Kit (Thermo Scientific ^TM^) following the manufacturer’s instructions.

The pellets were named as free-IgGs EVs from plasma and the supernatant were called sera without ICs and both were stored at −80 °C until used. The supernatant plasma without ICs was adjusted to pH 9.4 and used as describe above.

### ELISA

Nunc Immobilizer Amino 96 well plates (Thermo) were coated with 5 μg/well of synthetic MASP SP peptide diluted in (0.1 M) Bicarbonate/carbonate coating buffer pH 9.6, 1 h at RT or with an extract of total soluble proteins from E forms, obtained as described above. Next, the plates were washed three times with PBS-T, blocked with blocking buffer (skimmed milk 2% (V/W) in PBS-T) and incubated for 2 h at RT. Then, the wells were incubated with the different sera, with and without ICs, after the acid pH treatment and ultracentrifugation procedure (free ICs, FICs and ICs respectively), at the same dilution (1:100). Next, the wells were washed three times with PBS-T and incubated with HRP-conjugated secondary anti-human IgG antibody (sigma) (1:1000). After three PBS-T washes, the reaction was developed in peroxidase substrate (O-phenyl-diaminobenzidine plus 30% H_2_O_2_ (1 μl/ml) (Sigma-Aldrich) in 0.05 M phosphate-citrate buffer, pH 5.0) for 15 min at 27 °C. The reaction was stopped with a solution of H_2_SO_4_ (2N), and absorption measured at 492 nm in an ELISA Multiskan spectrum reader (Thermo). The negative controls were treated as the positive sera.

As control, pools of ten sera from healthy individuals from endemic countries were used to calculate the cut-off value using the formula: average of the OD values +3 × SD.

### Immune capture ELISA

The Immune capture ELISA was assayed in 96 well plates MaxiSorp (Nunc, Thermo Scientific^TM^) coated with a 4 μg/ml of anti- MASP SP rat immune sera in a volume of 100 μl/well and diluted in (0.1 M) bicarbonate-carbonate buffer (pH 9.6), overnight at 4 °C. After 3X washes with PBST, 300 μl/well of blocking buffer (skimmed milk 2% (V/W) in PBS-T) was added for 1 h at RT. Following 3X washes in PBST, 5 μg of ICs-free total protein from the different positive sera (CARD-Arr, CARD-ICC, DIG and ASYMP) were added in a final volume of 50 μl in PBS and incubated overnight at 37 °C (or EVs obtained from the Trypomastigote cultures depending of the experiments). After 3X washes with PBS and depending on the assay, the wells were incubated for 1 h at 37 °C with 5 μg/ml per well of anti-EVs-Trypo mouse immune sera, anti-TPTC rabbit immune sera, anti-CD9 antibody mouse immune sera (Biolegend), a dilution of positive sera (1:100) without ICs or PBS as negative control. After 3 washes with PBS, the plate was incubated with 100 μl of HRP-conjugated anti-mouse antibody (DAKO), HRP-anti-rabbit antibody (DAKO) or HRP-conjugated IgG anti-human (Sigma) as secondary antibodies at a dilution 1∶50000, 1:1000, 1:2000 and 1:1000 respectively in PBS for 1 h at RT. After the final 3 washes with PBS-T, the reaction was developed as described above.

### Complement inhibition by MASP SP and EVs

To evaluate the protection against complement lysis, the E forms were labelled with radiolabelled ^51^Cr as we previously described^19^.

To calculate the CH_50_ (50% cell lysis) 2.5 × 10^6^ labelled parasites/ml were incubated with different dilutions of complement (CHS [SIGMA]) and a pool of positive Chagas sera (inactivated by heating at 56 °C 30 min) and incubated at 37 °C for 1 h. The ^51^Cr release was measured in a γ-Spectrometer (Beckman, LS6000) using the following formulae: % LE = (CPMSbn.ctrl/CPSMSbn.Ctrl + CPMpelletCtrl) x 100; % LSP = (CPMSbn.Prb/CPSMSbn.Prb + CPMpelletPrb) x 100. Where: % LE = spontaneous liberation; % LSP = specific liberation; CPMSbn = CPM in supernatant; CPM pellet = CPM in the pellet; Ctrl = control; Prb = problem. The calculation of the complement DL50 parasite lysis (50 CHS) was obtained by lineal regression with the ^51^Cr release data of the different Complement dilutions using Graph Path InStat version 3.06 Software.

The inhibition complement assay was performed according to the methodology described by De Pablos *et al*.^19^. Briefly, once determined the 50 CHS, the labelled E-^51^Cr forms were treated, with a total of 1 μg/100 μl of synthetic MASP SP peptide and EVs from E, T and A forms of parasites and incubated with the inactivated sera. The percentage of lysis inhibition was calculated by the formula % IC = (LspC - Lsp/Lsc) x 100, where % IC is the percentage of complement inhibition.

### Statistical Analysis

The statistical analyses were performed by using the software GraphPad Instat v 3.05. The results were indicated as mean ± SD values of the different groups. The differences between concentrations of ICs in the different groups were analyzed by one-way Anova test, where p-value p ≤ 0.0001 (****) was considered to be extremely significant. Tukey-Kramer Multiple Comparisons Test was used to compare means between groups where p < 0.001(***) was considered extremely significant, p < 0.01(**) slightly significant and p > 0.05 (ns) considered as non-significant.

## Results

### Identification of EVs in *T. cruzi* PAN4 strain

**Figure 1:**
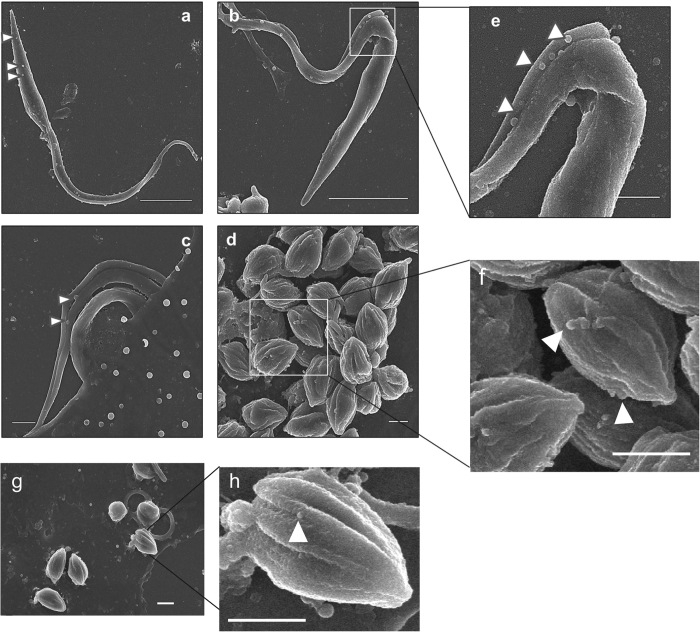
Release of EVs from the surface of *T. cruzi.* Culture derived epimastigote forms (**a,b**), Metacyclic trypomastigotes interacting with host mammalian cells (**c**) and free purified intracellular forms (**d–h**) were analyzed by scanning electron microscopy (SEM) (**e,f** and **h**) correspond to magnified image of (**b,d** and **g**) images. The arrows indicate released EVs. Size bar = 1 μm.

To confirm secretion of EVs to the culture medium in all forms of *T. cruzi* PAN4 strain (DTU I), the purified tissue-culture derived T and A forms, E and MT forms were incubated with free-EVs culture medium for 12 h and analyzed by SEM and TEM. The SEM analysis confirmed the existence of these vesicles on the parasite surface in all stages, as shown in Fig. 1. The T form showed several vesicles along its surface (Fig. 1a), being more abundant near the flagellar pocket (Fig. 1b,e). The MT forms infecting host cells and the A forms also showed secretion of EVs (Fig. 1c and d–h for the amastigote forms).

**Figure 2:**
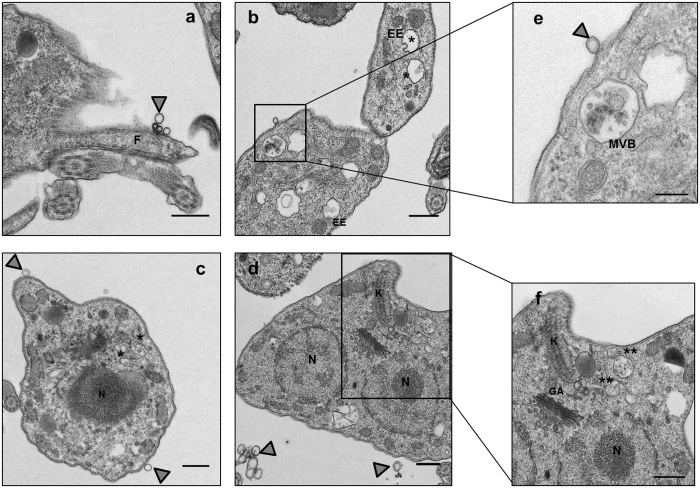
Formation and secretion of EVs in epimastigotes forms. Formation of multivesicular bodies (MVBs) and secretion of EVs by transmision electron microscopy (TEM). F: fagella; N: nucleus; EE: early endosome; MVB: multivesicular bodies; K: kinetoplastid. **Multivesicular bodies. Grey arrows: Released EVs Size bar = 500 nm.

TEM analysis corroborated the presence of outward secreted vesicles, budding from the plasmatic membrane and close to the flagellum (Fig. 2a). Moreover, typical multivesicular bodies (MVB) as well as early endosomes (EEs) in the process of MVB formation were observed in the parasite cytoplasm (Fig. 2b and e). Within the MVB, spherical membranous vesicles with a size between 50 to 100 nm (Exosomes size range) were observed. The localization of MVBs was close to the tubular network of the RE and Golgi, as shown in Fig. 2d and f.

### The MASP SP is transported in EVs

In the light of the recent results showing the presence of the C-term region of MASP released in EVs, we decided to check whether or not the MASP SP was also present in EVs.

**Figure 3:**
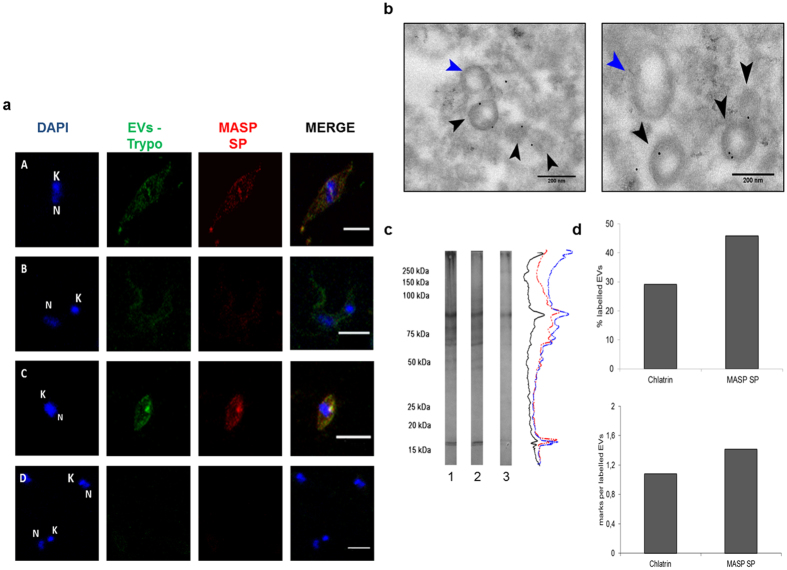
Presence of MASP SPs in EVs. (**a**) Immunolocalization of MASP SP and EVs in permeabilized trypomastigotes forms (A. epimastigotes,B.trypomastigotes, C.amastigotes, D. A co-incubation with mice and rat pre-inmune sera as negative controls in free permeabilized E forms) Size bar = 5 μm; N: nucleus; K:kinetoplast. (**b**). Immunogold labeling with 10 nm of gold particles of MASP SPs in purified EVs. Blue arrows: Non- labeled EVs. Black arrows: labeled EVs. (**c**) Western blot of ectosomes, exosomes and trypomastigote forms (line 1, 2 and 3 respectively) and their associated densitograms (red, blue and black lines respectively) using anti-EVs antibodies. (**d**) Quantification and comparison of labelled EVs using Chlatrin and MASP SP antibodies. Bar graphs representative of at least 100 EVs. Size bar = 200 nm.

We first analyzed the localization of MASP SP in E and T forms using confocal laser scanning microscopy with anti-MASP SP and anti-EVs antibodies (Fig. 3a). Both antibodies co-localized in E and A forms, and distributed irregularly in multiple foci near to the plasma membrane and the cytosol, with increasing signal close to the flagellar pocket in A forms. Although still co-localizing, the signal of MASP SP was less intense in T forms, when compared with EVs.

To analyze the MASP protein composition of *T.cruzi* EVs, a sample of EVs isolated from T forms and EVs (ecto and exosomes) secreted in parasite-host cell interaction medium (IM), were subjected to western blotting analysis using anti-MASP SP antibodies (Fig. 3b). For each EVs sample, the pattern of MASPs were similar, with proteins between ~75 to 25 kDa (with the exception of a ~160 kDa band), within the predicted size range for proteins belonging to the MASP family. The gold-immune labeling of the MASPs SP located this region at the surface and inside of T EVs (Fig. 3b), while no marks were detected when the T EVs were incubated with anti-CD9 antibodies as EVs human serum control (data not shown). The percentage of EVs labeled with Clathrin was 29.11% with 1 ± 0.2 mark per EVs, whereas a higher percentage was found for MASP SP (46%) in which a mean/average of 1.41 ± 0.80 marks per EVs was found, which represents an increase of ~63% for the MASP SP (Fig. 3d). The mean size of all EVs measured (n = 83) was 155 nm ± 6 nm.

### *T. cruzi*-derived EVs generate circulating ICs in patients with Chronic Chagas disease sera

**Figure 4:**
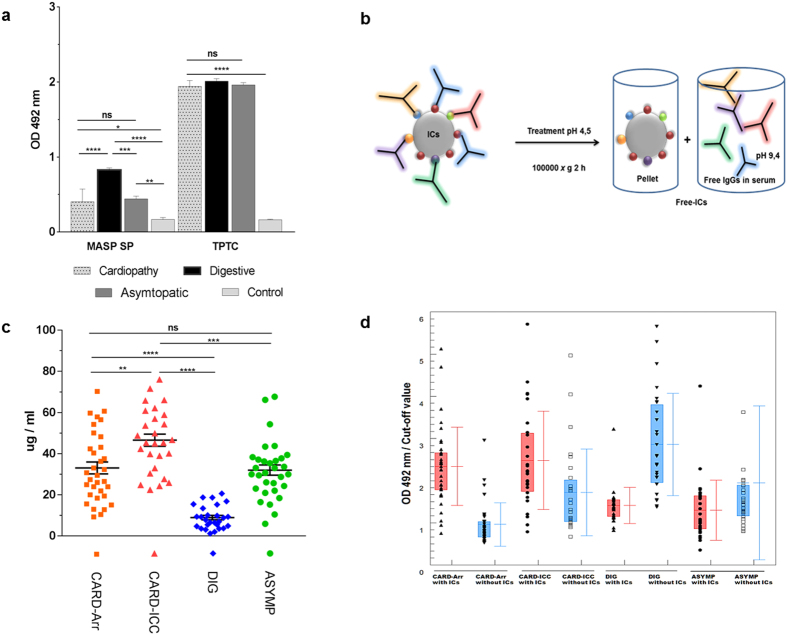
Humoral response against the MASP SP in patients with Chronic Chagas Disease. (**a**) IgG response against the MASP SP and soluble whole protein extract from *T. cruzi (*TPTC) of a pool of sera from argentinean Chagas disease patients classified as Asymtomatics, Cardiopathy, Digestive and non-infected control group from Bolivia and Argentina. Values with p-valor < 0.001(***) were considered as significantly different. (**b)** Graphical representation of the antibody-depletion of ICs using a treatment at low pH (4.5). **(c)** Protein concentrations of free- IgGs EVs (ug/ml) from each serum group after depletion of ICs. The horizontal bar represents mean values and the vertical bar represents SD values. Values with p-valor < 0.0001(****) were considered highly significant. (**d**) IgG response of each sera group before and after treatment at low pH (with and without ICs) against the MASP SP peptide. Turkey- Kramer test mutilple comparations was used. Values of p < 0.001(***) were considered as significantly different. Cut-off values were obtained calculating the mean of negative controls (sera of non-infected individuals) ± 3 (SEM).

The previous finding of the associated immune response against the conserved C-term MASP region, led us to study the immune response against MASP SP and its putative role in immune evasion. Firstly, we tested the antigenicity of the MASP SP synthetic peptide by ELISA against a pool of sera from patients with different pathologies (cardiopathy, digestive, asymptomatic and control), using an extract of total proteins from non-infective *T. cruzi* forms (TPTC) as a positive control. As a whole, the IgGs response against the peptide SP was positive for all tested sera but weaker compared to the response to TPTC (Fig. 4a). Despite being all positive, a significant difference was observed depending on the type of pathology. Indeed, the recognition of MASP SP was significantly higher in the digestive pool of sera (OD 0.82 ± 0.02) compared to cardiac (0.40 ± 0.17) or asymptomatic ones (OD 0.44 ± 0.03), whereas no significant differences were observed when TPTC was used as an antigen (Fig. 4a).

Next, we searched for the presence or absence of immune complexes (ICs) carrying MASP SPs in sera from patients with the different pathologies (Fig. 4b). After treatment, we corroborated the existence of proteins following the ultracentrifugation in the unbounded ICs pellets for each treated serum as shown in Fig. 4c. The median concentration of proteins present in ICs from sera was 8.91 ± 5.53 μg in digestive sera; 33.05 ± 16.62 μg in cardiac arrhythmia sera, 46.54 ± 15.23 μg in cardiac insufficiency sera and 32.01 ± 13.80 μg in the asymptomatic group sera. Overall, all groups showed statistically significant differences with respect to the digestive sera group. When the sera with and without ICs were tested against the peptide SP (Fig. 4d), the response in those without ICs was in accordance with the total IgGs previously assayed (Fig. 4a). When both types of sera were compared within each clinical group, patients with digestive symptoms without ICs (DIG non-ICs) showed the highest increase in response. As a whole, the sensitivity of the test for treated sera was 93% whilst that for untreated sera was 41%, specificity, being 99% in both cases.

**Figure 5:**
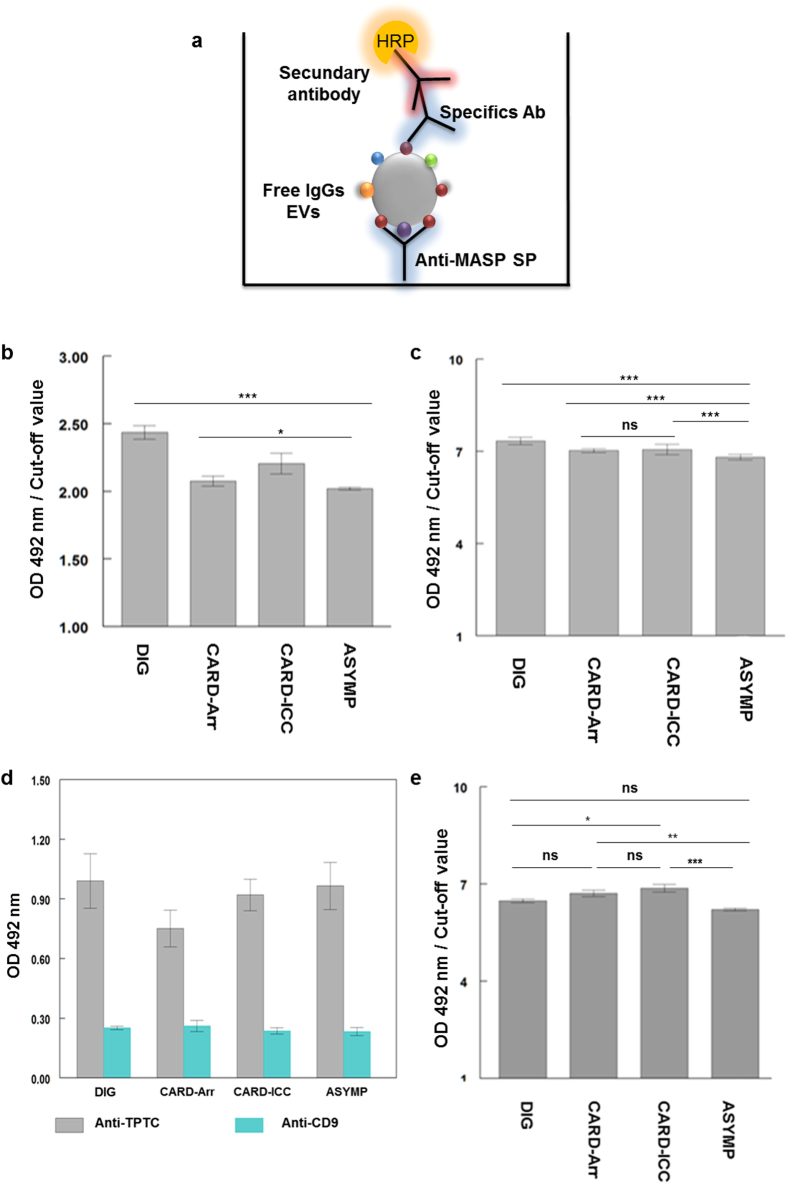
Detection of MASP proteins in ICs by immunocapture assay. **(a)** Schematic representation of the methodology followed in the immunocapture assays. **(b**) Detection of MASP SPs (capture antibody) in EVs from Chagas disease clinical group (target) using anti-EVs Trypo immunoglobulins (detection antibody) **(c).** Detection of free-IgGs EVs from a pool of each Chagas clinical sera group (target), using anti-SP MAPS as capture antibody and a dilution (1/100) of sera without ICs from Chagas disease clinical group as detection antibody. **(d)** *T.cruzi*-specificity of EVs in free-IgGs EVs using anti-TPTC immunoglobulin (*T. cruzi* positive control) and anti-CD9 antibodies (human positive control) as detection antibodies. (**e**) Detection of parasite EVs (target) in ICs by immunocapture assays using anti-MASP SP antibodies (captured antibody) and a dilution of a pool of chagasic patients’ sera (1/100) (detection antibody). Turkey- Kramer test multiple comparisons was used. Values of p < 0.001 (***) were considered as significantly different. Cut-off values were obtained calculating the mean of negative controls (sera of non-infected individuals) ±3 (SEM).

In order to determine if bloodstream trypomastigote secreted EVs were part of circulating ICs and whether or not they harbored MASP antigens on their surface, an immune capture assay was performed (Fig. 5a). The incubation of the pre-coated anti-MASP SP antibodies revealed a high response for the four clinical groups versus the healthy donors control group (cut-off value = 0.503), the group with digestive pathology being the most reactive of all (OD/cut-off =2.42) (Fig. 5b) and showing higher significance with respect the other groups (p < 0.001). When immune-capture ELISA assays were performed using pools of sera treated without ICs from each clinical group (dilution 1:100), the response was positive showing higher significance for all of groups (***) with respect to the digestive and the asymptomatic group, and non-significance (p > 0.05) between the two cardiopathic groups (CARD-Arr and CARD-ICC) (Fig. 5c). These results demonstrated the existence of MASP SP in ICs detected using anti-MASP SP specific immunoglobulins present in the immune sera, with the highest levels for the sera from digestive pathologies.

To corroborate the parasite origin of the ICs present in the sera, we used anti-TPTC control for parasite EVs and anti-CD9 antibody as a control of human EVs, keeping the anti-MASP SP antibodies as bait and using the above mentioned antigens for each clinical group. The findings obtained showed a high reactivity for *T. cruzi* antigens and a low response against anti-CD9 Abs. Therefore, this finding reveals that ICs captured by specific anti-MASP SP antibodies are of parasite origin, and not part of the human EVs present in human sera (Fig. 5d).

To determine if the EVs from *T. cruzi* form part of the of the ICs, the immune capture was slightly modified by using the same anti- MASP SP antibodies as bait but EVs excreted by the cultured T forms, as antigens. Next, the plate was incubated with the pool of patient’s sera and finally developed using an HRP conjugated anti-human IgG secondary antibody (Fig. 5e). The results showed that antibody recognition was very high with respect to the control group with significant differences between cardiac insufficiency and asymptomatic patients (p < 0.001), slightly significant differences between cardiac arrhythmic and asymptomatic (p < 0.01) and no significant differences (p > 0.05) between digestive and cardiac insufficiency patients (Fig. 5e).

**Figure 6:**
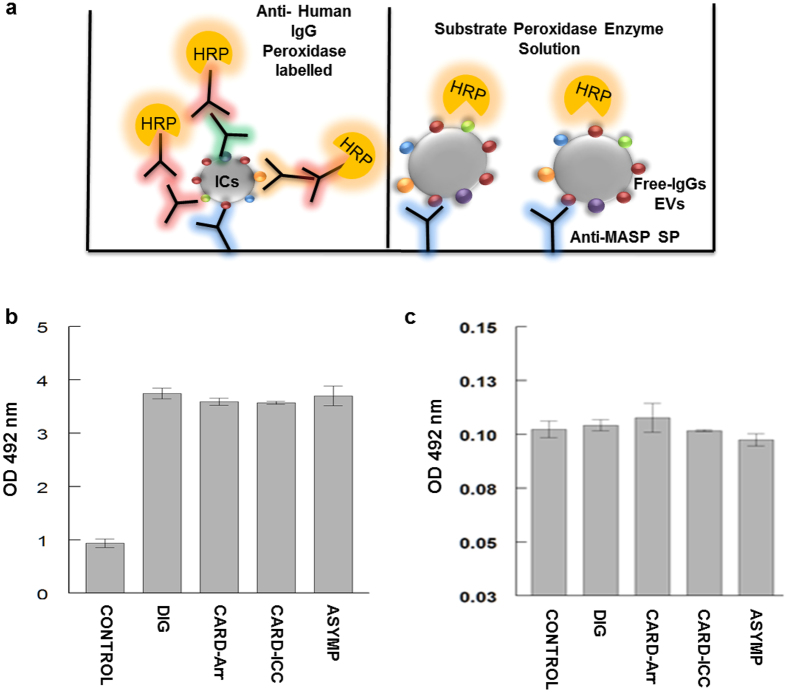
Control for the presence of immunoglobulin in ICs in human sera from chronic chagasic patients and for the absence of Peroxidase enzyme in EVs from ICs (**a**). Schematic representations of the methodology followed in the controls assays. (**b**) Determination of human immunoglobulins in ICs in sera from chagasic patients using HRP-conjugated IgG anti-human (Sigma) as detection antibodies (**c**). Determination of false positives in immunocapture assays by detection of Peroxidase enzyme in free-ICs EVs after low pH treatment. Anti-MASP SP was used as a capture antibody and revealed with the substrate solution for the HRP as detection.

To determine the presence of human immunoglobulins on the surface of the ICs, we purified the ICs from each pool of sera and coated them in the multi well plates. The plates were incubated with antihuman IgG secondary antibody conjugated to HRP. As shown in Fig. 6b, the absorbance of sera from the different clinical groups was ~2.5 higher than that of the controls, revealing that human immunoglobulins were present in the purified fractions.

Finally, a control assay was performed to determine the absence of enzyme peroxidase in the ICs. To do so we used the free-IgGs EVs from each sera group obtained by ultracentrifugation and incubated with the peroxidase substrate, as described above. The results showed that both control and patients’ samples lacked this enzyme in the ICs fraction, thus not interfering with the reactions (Fig. 6c).

### The MASP SPs and EVs from *T. cruzi* inhibits complement-mediated lysis

**Figure 7:**
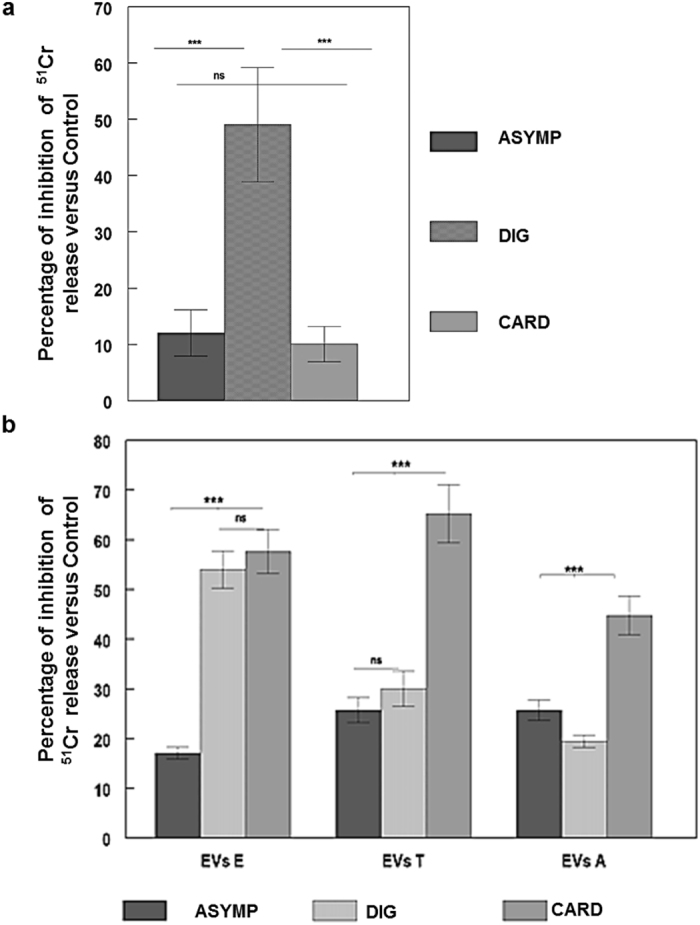
Complement inhibition assays. ^51^Cr labelled E, T or A forms were treated with 10% heat inactivated sera from chagasic patients diagnosed as digestive, cardiopathic or asymptomatic. After incubation with 1/18 dilution human complement (CH50) and 1 μg/100 μl of MASP SP peptide (**a**) and EVs from E, T and A forms (**b**) at 37 °C for 1 h. Non-inactivated pooled sera from chagasic patients was used as a positive control and a pool of inactivate sera without supplemented complement as a negative control.

To demonstrate the functionality in the immune evasion of the antigens released by EVs, a human complement inhibition assay was performed. The EVs and the MASP SP were able to inhibit the lysis mediated by the human complement system to a different degree in the different pools of positive sera (Fig. 7). In the case of MASP SP, the highest percentage of inhibition appeared in the digestive sera group with 50% of inhibition, with statistically significant differences (p < 0.001) with respect to the inhibition obtained for the cardiopathy and asymptomatic sera, where lower values (<20%) were detected (Fig. 7a). Using the same methodology, we tested the role of *T. cruzi* EVs in the immune evasion to the complement system in E and T forms. The highest inhibitory response to the complement lytic activity corresponded to sera from patients with cardiopathy (% inhibition >50%) (p < 0.001 among the sera pools) in EVs obtained from the three parasite forms, followed by digestive sera for the EVs secreted from E and T forms (>50% and 30% respectively). The lower protection in the three clinical groups was found for the EVs secreted from intracellular forms with respect to E or T EVs (Fig. 7b).

## Discussion

In eukaryotic cells, newly synthesized proteins that will be secreted or exposed on the external surface of cells have an N-terminus SP, which targets them to the ER^55^,^56^,^57^. Then, the SP is cleaved by a signal peptidase protease, releasing the polypeptide into the lumen of the ER^58^. In the present study, we have described for the first time, the presence of a MASP SP peptide in *T. cruzi* EVs, an alternative non-canonical pathway for SP secretion as well as the presence of an associated humoral response against this antigen region with the subsequent formation of circulating ICs.

The secretion of EVs by the different developing forms of *T. cruzi* was demonstrated by SEM (Fig. 1), and the presence of the MASP SP region ~30 amino acids of length including the cleavage site (29 position), in multiple non-processed MASP proteins was also confirmed by western blot, immunochemistry and confocal microscopy. According to the TEM observations and confocal microscopy (Figs 2 and 3), the parasites release EVs from different sources, either by budding of the plasma membrane (Ectosomes) or by the endocytic pathway with the consequent fusion of the MVBs with the plasma membrane followed by the release of exosomes. These data are in agreement with previous findings showing that the MASP C-term conserved region is also secreted via EVs^19^,^45^. Furthermore, the presence of early endosomes, MVBs with ILVs and ectosomes in epimastigote forms of *T. cruzi* was confirmed by TEM analysis (Fig. 2).

The secretome of *T. cruzi* is formed by different populations of vesicles together with soluble proteins involved in multiple processes such as metabolism, signaling, nucleic acid binding, and parasite survival and virulence pointing to the existence of different secretory/excretory pathways in the parasite^45^,^46^. One of the routes to prevent cells from proteotoxicity is by the formation of MVBs through maturation of early endosomes into late endosomes and the accumulation of ILVs. These MVBs are formed by inward budding of the early endosomal membrane. In this process many proteins, lipids and even cytosol are sequestered and specifically sorted^33^.

In *T. cruzi*, around 9% of total proteins released via EVs were predicted to be secreted by the classical pathways, while ~48% of proteins were secreted by non-classical pathways. Therefore, there is evidence that the secretome of *T. cruzi is* formed by proteins transported in EVs (ectosomes and exosomes)^44^,^45^,^59^, an alternative secretory pathway used by parasites for the secretion of unprocessed proteins^60^.

The present investigation is the first report showing the presence of SPs in EVs. The SPs normally harbor a basic amino terminal domain (N-domain), a hydrophobic medial domain (H-domain) and a polar domain that contain the cleavage site and produce the orientation for the peptidase cleavage^61^. The inefficiency of the cleavage depends on the sequence and protein misfolding. An excessive collision between proteins may lead to terminal misfolding and the frequency of protein interactions with the chaperones in the ER determines the folding rates^56^. The aberrant intracellular accumulation of unfolded proteins with non-cleavage SP might lead to the production of aggregasomes^62^ in the ER and Golgi, which have been observed in at least twenty human pathologies including Alzheimer’s, Parkinson’s diseases, the spongiform encephalopathy, type II diabetes or hypoparathyroidism^63^,^64^,^65^, cancer^66^, heart disease^67^ and even viral infection^68^. The accumulation in the ER of immature proteins due to the inefficient processing of the N-terminal region of cruzain cysteine protease has been also described in *T. cruzi*^69^,^70^. In this way, a similar process may be occurring with MASPs immature proteins bearing SPs that do not pass the RE quality control, and that will be consequently exported and secreted via EVs^69^,^70^. This theoretical processing error in MASP SP by the ER and subsequent release in EVs by trypomastigote forms may constitute a beneficial mechanism for the establishment of the parasite in the mammalian host, contributing to its evasion of the immune response.

The release of EVs has been linked to parasite immune evasion^44^,^71^. For instance, *T. cruzi* trypomastigotes induce the secretion of host plasma-membrane derived vesicles which results in an inhibition of complement mediated death, therefore facilitating infection by the parasite as well as activating the pro-inflammatory response in the host cell^49^,^59^. The infective forms express a surface coat of mucins and *trans*-sialidases which have been also described as part of the EVs protein cargoes and protect the parasite from complement-mediated lysis^20^,^21^,^22^,^45^,^72^,^73^.

In this study, we have detected the humoral immune response against MASP SP of sera pools from different clinical groups of patients with Chagas disease as well as the triggering of lytic antibodies, as demonstrated by complement inhibition assays, the group with digestive pathology being the most reactive (~50% of inhibition) (Fig. 7). The immunoglobulin depletion from ICs of individual sera showed the presence of a higher antibody response against the MASP SP region in patients with digestive pathology (Fig. 5), confirming the presence of circulating ICs in Chagas disease patients as previously described^25^,^26^. Digestive anomalies are present in only 8–10% of Chagas disease patients and are characterized by a dilation of esophagus and/or colon (mega esophagus and/or mega colon) and also damage in the peripheral nerves. This might be due to either an increased antibody production and proliferative responses to peripheral myelin components^72^ or as a consequence of the damage due to deposits of ICs in the nerve tissue, as reported for other infectious diseases, such as filariasis and schistosomiasis^74^.

As confirmed by immune capture assays, the ICs present in sera are formed by EVs carrying the MASP SP (Fig. 6). Recently, Ohyama *et al*.^30^ identified 39*T. cruzi* antigens in circulating ICs including surface antigens such as *trans*-sialidases, gp63 and MASPs in samples from patients with Chagas disease. Similarly to the findings of our study, the same authors also showed the association of two hypothetical proteins with ICs from patients suffering mega colon^30^, revealing an association between ICs composition and chronic Chagas disease manifestations.

The humoral response to MASP SP region might be due to the presence of numerous MASP members in released EVs (ectosomes and exosomes) during the course of the infection^19^ (Fig. 3b). Likewise, the composition of the EVs released by E, T and A forms and its associated immune response must be highly dynamic and variable in its levels as revealed by the different inhibition of the complement system when using different sources of sera and EVs (Fig. 7b).

Therefore, it could be hypothesized that the existence of a battery of different MASP proteins with differential expression within cellular populations of *T. cruzi*^14^,^75^ following different routes; i) to be part of the surface coat and play a role in the host cell invasion together with other surface proteins^16^,^17^, ii) are individually secreted in the extracellular space or as a part of secreted/excreted pathways by EVs, which would represent mechanisms of immune evasion as demonstrated by complement inhibition assays^19^.

In conclusion, we have demonstrated the secretion of the MASP conserved SP region in trypomastigote EVs that could act as an antigen transporting mechanism, thus promoting parasite evasion of the host immune system. The formation of ICs with EVs as an antigenic source, would favor the capture of EVs through FC receptors of host macrophages, modulating the immune response of these cells against the parasite and possibly promoting the expansion and survival of *T. cruzi* in infected host cells. Moreover, the detection of MASP surface protein family members in EVs would constitute a potentially valuable biomarker for the differential diagnosis or prognosis of chronic Chagas disease, in particular in patients with mega visceral manifestations.

## Additional Information

**How to cite this article:** Díaz Lozano, I. M. *et al*. Immune complexes in chronic Chagas disease patients are formed by exovesicles from *Trypanosoma cruzi* carrying the conserved MASP N-terminal region. *Sci. Rep.* **7**, 44451; doi: 10.1038/srep44451 (2017).

**Publisher's note:** Springer Nature remains neutral with regard to jurisdictional claims in published maps and institutional affiliations.

## Supplementary Material

Supplementary Information
