# Supplementary material for: Immune complexes in chronic Chagas disease patients are formed by exovesicles from Trypanosoma cruzi carrying the conserved MASP N-terminal region
Source: Sci Rep. 2017 Mar 15;7:44451. doi: 10.1038/srep44451 (PMC5353755; doi:10.1038/srep44451)
Supplement: Supplementary Information [file srep44451-s1.pdf]

Supplementary Files of

Manuscript Title: Immune complexes in chronic Chagas disease patients are formed by exovesicles from *Trypanosoma cruzi* carrying the conserved MASP N-terminal region.

Authors: Isabel María Díaz Lozano[1], Luis Miguel De Pablos[1,2], Silvia Andrea Longhi[3], María Paola Zago[4], Alejandro Gabriel Schijman[3] and Antonio Osuna[1].

[1]Departamento de Parasitología, Grupo de Bioquímica y Parasitología Molecular, Campus de Fuentenueva, Universidad de Granada, 18071 Granada, Spain.

[2] Centre for Immunology and Infection (CII), Biology Department, University of York, York, UK

[3]LabMeCh, INGEBI-CONICET, Buenos Aires, Argentina.

[4]Instituto de Patología Experimental (IPE) CONICET-UNSa, Argentina.

*Author for correspondence: Antonio Osuna.

Institute of Biotechnology, University of Granada, Campus Universitario Fuentenueva, 18071 Granada, Spain.

Supplementary figure 1. Structure of synthetic peptide MASP SP with four branches bound by its Lysine residues.

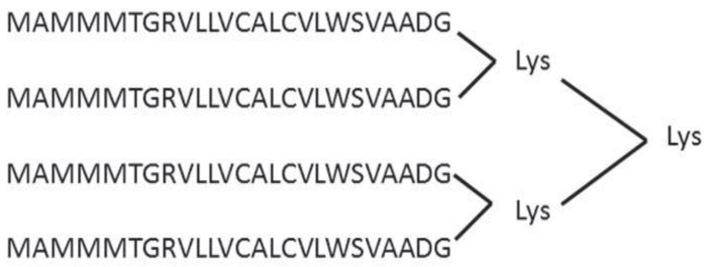

Table S1: Clinical characteristics of patients with demonstrable cardiomyopathy

	CARD-Arr (n=36)	CARD-ICC (n=28)
Mean age (SD)	59 (11)	62 (13)
Mean heart rate (SD), *beats/min*	64 (12)	67 (10)
**Electrocardiographic findings**		
Atrioventricular block, n (%)	9 (25.0)	8 (28.7)
Right bundle branch block, n (%)	10 (27.7)	15 (53.6)
Left bundle branch block, n (%)	1 (2.7)	2 (7.1)
Left anterior hemiblock, n (%)	6 (16.7)	13 (46.4)
**24-hour Holter monitoring**		
Supraventricular extrasystole, n (%)	7 (19.4)	5 (17.9)
Supraventricular tachycardia, n (%)	13 (36.1)	11 (39.3)
Ventricular extrasystole, n (%)	15 (41.7)	22 (78.6)
Ventricular tachycardia, n (%)	4 (11.1)	13 ((46.4)
**Pacemaker/implanted cardio-defibrillator, n (%)**	20 (64.5)	20 (71.4)
**Main echocardiographic characteristics**		
Mean left ventricular end-diastolic diameter (SD), *mm*	48 (4.5)	63.8 (7.8)
Left ventricular systolic function (n)	Conserved (36)	Severe (19)<br>Moderate (5)<br>Mild (4)

Chronic Chagas heart disease patients were subclassified according to Consensus Statement on Chagas-Mazza Disease (REV. ARG. CARDIOL 79 (6): 544-564, 2011). **CARD-Arr**: patients with one or more arrhythmias and/or conduction disorders in ECG and/or Holter monitoring, **CARD-ICC**: patients with heart failure, cardiomegaly and/or

ventricular dysfunction (left ventricular end-diastolic diameter > 55 mm), with or without arrhythmias.
